# Macadamia nut effects on cardiometabolic risk factors: a randomised trial

**DOI:** 10.1017/jns.2023.39

**Published:** 2023-05-08

**Authors:** Julie L. Jones, Joan Sabaté, Celine Heskey, Keiji Oda, Fayth Miles, Sujatha Rajaram

**Affiliations:** School of Public Health, Loma Linda University, Loma Linda, CA, USA

**Keywords:** Adiposity, Body composition, Cholesterol, LDL-C, Macadamia nuts, Palmitoleic acid, Apo A1, apoprotein A1, Apo B, apoprotein B, BFM, body fat mass, CV, coefficient of variation, CVD, cardiovascular disease, DLM, dry lean mass, HOMA2, homeostasis model assessment 2, IR, insulin resistance, LBM, lean body mass, Mac, macadamia nuts, oxLDL, oxidised LDL, % Body fat, percent body fat, sdLDL, small dense low-density lipoprotein, se, standard error, sem, standard error of the mean, SMM, skeletal muscle mass, TAG, triacylglycerol, TBW, total body water, TC, total cholesterol, TEE, total energy expenditure, VLDL,, very low-density lipoprotein, WC, waist circumference

## Abstract

We sought to examine the effects of daily consumption of macadamia nuts on body weight and composition, plasma lipids and glycaemic parameters in a free-living environment in overweight and obese adults at elevated cardiometabolic risk. Utilising a randomised cross-over design, thirty-five adults with abdominal obesity consumed their usual diet plus macadamia nuts (~15 % of daily calories) for 8 weeks (intervention) and their usual diet without nuts for 8 weeks (control), with a 2-week washout. Body composition was determined by bioelectrical impedance; dietary intake was assessed with 24-h dietary recalls. Consumption of macadamia nuts led to increased total fat and MUFA intake while SFA intake was unaltered. With mixed model regression analysis, no significant changes in mean weight, BMI, waist circumference, percent body fat or glycaemic parameters, and non-significant reductions in plasma total cholesterol of 2⋅1 % (−4⋅3 mg/dl; 95 % CI −14⋅8, 6⋅1) and low-density lipoprotein (LDL-C) of 4 % (−4⋅7 mg/dl; 95 % CI −14⋅3, 4⋅8) were observed. Cholesterol-lowering effects were modified by adiposity: greater lipid lowering occurred in those with overweight *v.* obesity, and in those with less than the median percent body fat. Daily consumption of macadamia nuts does not lead to gains in weight or body fat under free-living conditions in overweight or obese adults; non-significant cholesterol lowering occurred without altering saturated fat intake of similar magnitude to cholesterol lowering seen with other nuts.

Clinical Trial Registry Number and Website: NCT03801837 https://clinicaltrials.gov/ct2/show/NCT03801837?term = macadamia + nut&draw = 2&rank = 1

## Introduction

Nuts are now considered part of a heart-healthy diet, having been associated with a reduced risk of cardiovascular disease (CVD) and death in several large epidemiologic studies^(1–4)^ and a multicenter clinical trial^(5)^. One serving (28 g) of nuts per day is thought to reduce coronary heart disease by 29 % and CVD by 21 %^(6)^. The benefits of nut intake are at least partially related to their plasma lipid-lowering effects. A meta-analysis of the effects of tree nuts found a significant reduction in total (TC) (−4⋅7 mg/dl), LDL-C (−4⋅8 mg/dl), apoprotein (Apo) B (−3⋅7 mg/dl) and triacylglycerol (TAG) (−2⋅2 mg/dl)^(7)^. Importantly, the health benefits of nuts occur without an increase in body weight, body mass index (BMI), body fat or waist circumference (WC), despite their high fat content^(8–13)^. Evidence is accumulating, however, that the lipid-lowering effects of dietary interventions, including nuts, are lower in the setting of overweight/obesity or insulin resistance (IR)^(14–17)^. In our pooled analysis of nut consumption in twenty-five intervention trials^(14)^, the cholesterol-lowering effects of nuts were greatest in participants with a normal weight (BMI < 25 kg/m^2^), and progressively lower, though still significant, in those with overweight and obesity.

Macadamia nuts are a unique yet little-studied nut indigenous to the eastern shores of Australia. They contain the largest percent of both total fat and monounsaturated fat (MUFA) of edible nuts: 75 % of energy from fat, most of which is MUFA (82 %); very little polyunsaturated fat (PUFA) and 12–18 % saturated fat (SFA)^(18,19)^. The MUFA makeup is distinctive in that it is 17–19 % palmitoleic acid, and most of the remainder is oleic acid. Besides macadamia nuts, palmitoleic acid is present in very few foods in significant amounts (sea buckthorn oil is an exception), though it is produced endogenously in the liver and adipose tissue^(20)^. Data are emerging that adipose-derived palmitoleate may function as a beneficial lipokine, reducing hepatic *de novo* lipogenesis, IR and adiposity^(20–22)^. This would be expected to improve metabolic dyslipidemia, resulting in lower TAG and small dense LDL-C (sdLDL), and higher HDL-C, particularly in the setting of visceral adiposity (and greater metabolic dyslipidemia)^(23,24)^.

To date, five small studies have found that macadamia-containing diets lower TC and LDL-C and have variable effects on TAG and HDL-C^(25–29)^. However, three of four studies that reported macronutrient intakes were controlled feeding studies confounded by a purposeful reduction in SFA in the macadamia diets (reductions of 5–6 % of energy) compared to the reference diet^(25,28,29)^. The effects of macadamia nuts in free-living conditions without a reduction in SFA are unclear. Additionally, none of the studies examined body composition, glycaemic variables or atherogenic lipoproteins (sdLDL and oxidised LDL-C (oxLDL)), and four of the five studies focused on normal and overweight individuals; only one study included participants with a BMI ≥ 30 kg/m^2(25)^. No prior studies of macadamia nuts have considered lipid effect modifications by adiposity or IR. In three studies that examined body weight, two reported a small but significant lowering with macadamia nuts^(26,27)^, while no change was seen in the third^(29)^.

Thus, we aimed to determine the impact of daily consumption of macadamia nuts on body weight and composition, as well as plasma lipids and glycaemic variables, in a free-living environment in overweight and obese adults at elevated cardiometabolic risk. We hypothesised that macadamia nut consumption would result in stable or reduced weight, reduced TC, LDL-C and the components of metabolic dyslipidemia (pre-specified primary outcomes), and that adiposity and/or IR would be effect modifiers of the cholesterol-lowering effects (pre-specified secondary outcomes).

## Methods

### Study design

The present study, referred to as the MAC Study (Macadamia Nut Effects on Adiposity and Cardiovascular Risk Factors), was a randomised controlled 2 × 2 cross-over study in free-living adults consuming their habitual diet, with and without macadamia nuts as 15 % of daily energy. Each diet was consumed for 8 weeks with a 2-week washout period. All recruitment and data collection activities took place at the Nutrition Research Center (NRC) at Loma Linda University, Loma Linda, California.

### Participants

Participants were recruited from Loma Linda and surrounding communities via a multistage selection process. Following the completion of an online screening questionnaire, potential participants were invited for a group information session. Candidates then underwent a secondary screening that included basic anthropometric measurements and an interview by study researchers to determine eligibility. Study enrolment was done by study authors and NRC staff.

Eligible participants were overweight and obese men and women with abdominal obesity as measured by an elevated WC and at least one additional cardiometabolic risk factor. Specifically, eligibility criteria included men and postmenopausal women aged 40–75 with a BMI of 25–39 kg/m^2^, a waist WC ≥ 88⋅9 cm (35 in) for women and ≥ 101⋅6 cm (40 in) for men^(30)^, and ≥one additional cardiometabolic risk factor (plasma TC ≥ 200 mg/dl, LDL-C ≥ 100 mg/dl, TAG ≥ 150 mg/dl, fasting glucose ≥100 mg/dl, or hypertension defined as blood pressure ≥130/85 mmHg or current use of anti-hypertensive medication).

Exclusion criteria included nut allergies; medications affecting lipid or glucose metabolism or immune modulators; high doses of lipid-lowering dietary supplements in the last 6 months; the presence of significant chronic disease including diabetes; cancer in the last 10 years (excluding non-melanoma skin cancer); a weight change of >10 % of body weight in the prior 3 months; high alcohol intake (>1 drink/d for women, >2 drinks/d for men) and tobacco use.

We calculated the sample size to provide 80 % power to detect a 10 mg/dl change in LDL-C, with an *α* of 0⋅05, and a correlation of 0⋅9, allowing for a 15 % dropout rate. Randomisation was done utilising a random numbers table by study authors.

This study was conducted according to the guidelines laid down in the Declaration of Helsinki and all procedures involving human participants were approved by the Institutional Review Board at Loma Linda University. Written informed consent was obtained from all participants prior to beginning the study. The trial is registered at ClinicalTrials.gov, NCT03801837.

### Dietary intervention

Macadamia nuts were provided as 15 % of estimated total energy expenditure (TEE) in pre-measured daily portions during the intervention period. Daily energy requirements were estimated using the Mifflin St-Jeor equation recommended for use in overweight and obese participants^(31)^ adjusted by a factor for physical activity assessed with the Rapid Assessment of Physical Activity (RAPA) form^(32)^. The estimated TEE was then used to assign each participant to a level of macadamia nut intake. Potential energy intakes were bundled into groups spanning 200–300 kcal, and the mean of each was used to calculate 15 % of kcal to be provided as macadamia nuts. This resulted in four groups of nut intake: 35, 42, 48 and 59 g.

Participants met with a study clinician every other week throughout the study. All participants were asked to consume their habitual diet, with macadamia nuts replacing calories approximately equal to their allotment of macadamia nuts during the intervention period, to avoid all other nuts, and to limit seeds to ≤2 times per week. During the control phase, participants were asked to continue their habitual diet but abstain from all nuts and limit seed intake.

### Data collection

Anthropometrics including height, weight, body composition and WC were measured at baseline and at the end of each diet period. Height was measured using a digital stadiometer (Prodoc Detecto, Webb City, MO). WC was measured with a Gulick measuring tape placed 1 cm above the navel. Weight and body composition were measured with an InBody device (InBody570, Seoul, Korea). Participants were asked to fast and avoid exercise for 12 h, abstain from water or other beverages for 4 h and avoid alcohol for 48 h prior to each assessment. Pre-specified body composition variables included body fat mass (BFM), % body fat, skeletal muscle mass (SMM), lean body mass (LBM), dry lean mass (DLM) and total body water (TBW). LBM is the sum of DLM and TBW; LBM plus BFM is equal to body weight.

Fasting blood samples were collected at baseline and at the end of each diet period. Blood samples were immediately processed, and aliquoted plasma was stored at −80°C. Laboratory analyses were performed at the Jean Mayer USDA Human Nutrition Research Center on Aging at Tufts University. Glucose, total cholesterol, triglycerides, sdLDL, HDL-C, ApoA1 and ApoB were measured on an AU480 Clinical Chemistry Analyzer (Beckman Coulter, Inc., Diagnostics Division Headquarters, Brea, CA) as previously described^(33–37)^. LDL-C and VLDL were calculated using the Friedewald equation^(38,39)^. Oxidised LDL was measured with a sandwich enzyme-linked immunosorbent assay (Human Oxidized LDL ELISA Kit, Novus Biologicals, LLC Centennial, CO 80112) on the BioTek Instrument ELx 808 Microplate Reader. Insulin was measured using the IMMULITE 2000^(40)^. Serum fatty acids were quantified by gas chromatography with a flame ionising detector (Autosystem XL gas chromatograph, Perkin Elmer, Boston, MA). Peaks of interest were identified by comparison with authentic fatty acid standards (Nu-Chek Prep, Inc. Elysian, MN), and expressed as molar percentage (mol%) proportions of total fatty acids^(41–44)^.

Intra- and inter-assay % coefficients of variation (CV) for total cholesterol were 2⋅0 and 2⋅8 %. For triglycerides, intra- and inter-assay CV were 2⋅0 and 3⋅4 %, and for HDL-C, they were 3⋅0 and 5⋅0 %. For sdLDL, the average intra- and inter-assay CV were less than 3⋅5 and 5⋅0 %. For oxLDL, the average intra- and inter-assay CV were less than 4⋅7 and 5⋅3 %, and for ApoA1 and ApoB, they were less than 2⋅5 and 3⋅5 %. For fatty acids, inter-assay CV was <4⋅5 % for fatty acids present at levels >1 mol%. Intra-assay CV for fatty acids varied by concentration: 1⋅3–2⋅4 % for fatty acids present at >5 mol%, 1⋅9–3⋅5 % for fatty acids present at levels between 1 and 5 %, 1⋅4–11⋅8 % for those present at 0⋅5 to <1 %, and 8⋅2–12⋅2 % for fatty acids present at <0⋅05 mol%.

The Homeostasis Model Assessment calculator version 2 (HOMA2) (The Oxford Center for Diabetes, Endocrinology and Metabolism, Oxford, UK) was used to quantify HOMA2-IR values^(45)^. Fasting insulin and glucose values were inputted into the HOMA2 calculator to determine HOMA2-IR values for each subject. The correlation between the HOMA calculator and the euglycaemic clamp method is approximately 0⋅70–0⋅88^(46)^.

Dietary intake was monitored with 24-h dietary recalls using the multiple-pass method with Nutrition Data System for software version 2018 (University of Minnesota, Minneapolis, MN). Six recalls were obtained per subject: two at baseline and two during each diet period (one weekday and one weekend day per phase). Recalls with unrealistic intakes <500 kcal or >4100 kcal were excluded from analysis. Energy and macronutrient intakes were averaged for each phase. Intake of macadamia nuts was also documented daily in a diary provided to participants.

Compliance was monitored at bi-weekly visits by review of subject diaries, allowing us to rapidly address any issues. We assessed compliance with the 24-h diet recalls and an objective biomarker (plasma palmitoleic acid).

### Data analysis

Statistical analyses were performed with SPSS Statistics version 28 and R version 4.1.2. Baseline data was evaluated with and without dropouts with independent sample *t*-tests for quantitative data and Pearson *χ*^2^ tests for categorical data to assess randomisation between groups. Endpoint data was evaluated using a mixed-effects regression model, with treatment, sequence and period as fixed-effect terms and participants as a random-effects term. Estimated marginal means with standard error of the mean (sem), mean differences with standard error of the difference (sed), *P*-values and 95 % confidence intervals (CIs) of the difference are presented. Additional analyses of anthropometric variables with inclusion of energy intake and baseline percent body fat produced similar results, and these covariates were not included in subsequent models.

To examine whether TC and LDL-C effects were modified by adiposity (pre-specified secondary outcomes), we performed subgroup analyses with dichotomised variables for BMI (25–29⋅9 *v.* ≥30 kg/m^2^), WC (<108 cm *v.* ≥108 cm, the cohort median) and % body fat (<43 % or ≥43 %, the cohort median). The dichotomised variables were added to the above model as fixed effects and outcomes were evaluated by treatment (macadamia nuts *v.* control). *P*-values for the interactions between subgroups and treatment are presented.

Data analysis was performed ‘per protocol’. Three participants dropped out before any endpoint data was collected and were not included in the analyses. Independent sample *t*-tests comparing ‘completers’ to ‘non-completers’ showed no differences in weight, BMI, WC, TC or LDL-C, though the non-completers were younger and appeared to have more severe metabolic syndrome with higher TAG and sdLDL, and lower HDL-C. The non-completers also consumed less energy, total fat, SFA, MUFA and PUFA at baseline.

All lipid variables were normally distributed except VLDL, TAG, insulin and HOMA2-IR. The base 10 logarithm of these variables were normally distributed. Analyses utilising the logarithm of VLDL and TAG did not differ from that of the primary variables. Insulin and HOMA2-IR were log-transformed and back-transformed estimated marginal means are presented.

## Results

Thirty-eight participants initially enrolled. Three participants dropped out before completion of the first period (two for family emergencies; one subject developed a gallbladder disorder while in the macadamia period, possibly due to fat intolerance, and was advised by her physician to withdraw). A total of thirty-five participants completed the study and were available for analysis. Baseline characteristics are presented in Table 1. There were no differences between randomised groups in baseline anthropometrics, nutrient intake, plasma fatty acids, plasma lipids or glycaemic variables. Cardiovascular risk factors were equally distributed with the exception of LDL-C; more participants who began in the control group had an LDL-C ≥100 mg/dl. Twenty percent of the participants were male. Approximately half (*n* 18) were overweight (BMI 25–29⋅9 kg/m^2^), and half (*n* 17) were classified as obese (BMI ≥ 30 kg/m^2^).

**Table 1.** Baseline characteristics for all participants and by randomised order of treatment

	All Participants *n* 35		Mac → Control *n* 18		Control → Mac *n* 17		*P*-value
Gender^a^	28 F, 7 M		15 F, 3 M		13 F, 4 M		0⋅61
Age and Anthropometrics^b^	Mean	sd	Mean	sd	Mean	sd	

Age (y)	62⋅1	8⋅2	62⋅4	7⋅8	61⋅0	9⋅1	0⋅63
Weight (kg)	83⋅3	14⋅1	84⋅5	14⋅7	82⋅0	13⋅7	0⋅61
BMI (kg/m^2^)	30⋅3	3⋅4	30⋅2	3⋅7	30⋅4	3⋅3	0⋅905
Waist Circumference (cm)	107⋅5	9⋅5	108⋅1	10⋅7	106⋅8	8⋅3	0⋅69
Body fat (%)	41⋅9	5⋅9	41⋅7	5⋅4	42⋅1	6⋅5	0⋅83

Lipids and glycaemic variables^b^	Mean	sd	Mean	sd	Mean	sd	

Total Cholesterol mg/dL	204⋅2	29⋅6	199⋅2	29⋅4	209⋅5	29⋅9	0⋅31
LDL-C mg/dL	121⋅0	27⋅7	114⋅6	27⋅5	127⋅8	27⋅1	0⋅16
HDL-C mg/dL	56⋅8	11⋅4	56⋅7	9⋅3	56⋅9	13⋅7	0⋅95
TAG mg/dL	132⋅0	51⋅7	139⋅9	50⋅4	123⋅6	53⋅2	0⋅36
sdLDL mg/dL	39⋅2	11⋅1	35⋅9	12⋅1	42⋅6	9⋅2	0⋅70
oxLDL ng/mL	55⋅3	27⋅7	57⋅9	30⋅1	52⋅4	25⋅6	0⋅56
Glucose mg/dL	103⋅8	7⋅3	104⋅6	8⋅2	103⋅1	6⋅5	0⋅55
Insulin mIU/mL	11⋅7	5⋅8	13⋅3	6⋅0	9⋅9	5⋅1	0⋅80
HOMA2-IR	1⋅4	0⋅7	1⋅5	0⋅7	1⋅1	0⋅6	0⋅08

CV Risk Factors – *n* (%)^a^	n	%	n	%	n	%	

Total Cholesterol ≥ 200 mg/dl	23	66	12	67	11	65	0⋅90
LDL-C ≥ 100 mg/dl	28	80	12	67	16	94	0⋅04
TAG ≥ 150 mg/dl	9	26	6	33	3	18	0⋅29
HDL-C ♀ <50 mg/dl; ♂<40 mg/dl	7	20	2	11	5	29	0⋅18
Fasting glucose ≥ 100 mg/dl	24	69	13	72	11	65	0⋅63
BP ≥ 130/85 or BP medication	20	57	10	56	10	59	0⋅845
WC ♀ ≥35 in, ♂ ≥40 in	35	100	18	100	17	100	NA
Metabolic Syndrome *n* (%)	18	51	9	50	9	53	0⋅86

Mac, macadamia nuts; sd, standard deviation; TAG, triacylglycerol; sdLDL, small dense LDL; oxLDL, oxidised LDL; HOMA2-IR, Homeostasis Model Assessment (version 2)-Insulin Resistance; BP, blood pressure; WC, waist circumference.

a Pearson *χ*^2^ test.

b Independent sample *t*-test.

Mean daily energy intake was slightly higher during the macadamia nut phase (~97 kcal/d; NS) (Table 2). Macronutrient intakes between the macadamia and control diet phases differed primarily in total fat and MUFA intake. Total fat was an average of 26 g/d higher with macadamia nuts (*P* < 0⋅001; 95 % CI 13⋅2, 38⋅8), with most of the difference attributable to MUFA, which was 24⋅7 g/d higher with macadamia nuts (*P* < 0⋅001; 95 % CI 19⋅7, 29⋅6), as expected. Mean SFA intake was slightly higher (2⋅6 g/d; NS), while mean PUFA, protein and carbohydrate intakes were all slightly lower during the macadamia nut phase. The PUFA:SFA ratio (P:S ratio) was nearly 20 % lower with macadamia nut consumption (*P* = 0⋅01; 95 % CI −0⋅3, −0⋅045). Energy-controlled nutrient analyses were similar. Compliance with macadamia nut consumption as measured by inclusion in 24-h dietary recalls was 94 %.

**Table 2.** Dietary intakes at baseline and during macadamia nut and control diets^a^

	Baseline		Mac		Control		Mac–Control			
	Mean	% Energy	Mean	% Energy	Mean	% Energy	Mean Diff	sed	95 % CI	*P*-value
Energy (kcal)	1674		1849		1753		96⋅9	106⋅9	−120⋅6, 314⋅4	0⋅37
Protein (g)	69⋅4	16⋅5 %	64⋅8	13⋅8 %	68⋅4	15⋅8 %	−3⋅7	4⋅7	−13⋅2, 5⋅9	0⋅44
Carbohydrate (g)	192⋅0	45⋅7 %	192⋅6	40⋅9 %	209⋅2	48⋅4 %	−16⋅7	15⋅2	−47⋅5, 14⋅2	0⋅28
Fat (g)	70⋅6	37⋅8 %	94⋅7	45⋅3 %	68⋅7	35⋅8 %	26⋅0	6⋅3	13⋅2, 38⋅8	<0⋅001
SFA (g)	21⋅5	11⋅5 %	26⋅1	12⋅5 %	23⋅5	12⋅2 %	2⋅6	2⋅4	−2⋅2, 7⋅4	0⋅28
MUFA (g)	25⋅1	13⋅4 %	47⋅0	22⋅5 %	22⋅3	11⋅6 %	24⋅7	2⋅4	19⋅7, 29⋅6	<0⋅001
PUFA (g)	18⋅0	9⋅6 %	14⋅7	7⋅0 %	16⋅6	8⋅6 %	−1⋅9	1⋅9	−5⋅8, 1⋅9	0⋅32
P:S ratio	0⋅84	−	0⋅56	−	0⋅75	−	−0⋅187	−	−0⋅3, −0⋅045	0⋅01
Fibre (g)	20⋅6	−	21⋅5	−	19⋅9	−	1⋅5	1⋅6	−1⋅7, 4⋅8	0⋅34
Cholesterol (mg)	288⋅2	−	247⋅0	−	283⋅2	−	−36⋅2	37⋅4	−112⋅3, 39⋅9	0⋅34

Mac, macadamia nut; Mean Diff, mean difference; sed, standard error of the difference; SFA, saturated fatty acids; MUFA, monounsaturated fatty acids; PUFA, polyunsaturated fatty acids; P:S, polyunsaturated to saturated fatty acid ratio.

a Mixed model regression controlled for sequence and period.

**Fig. 1.**
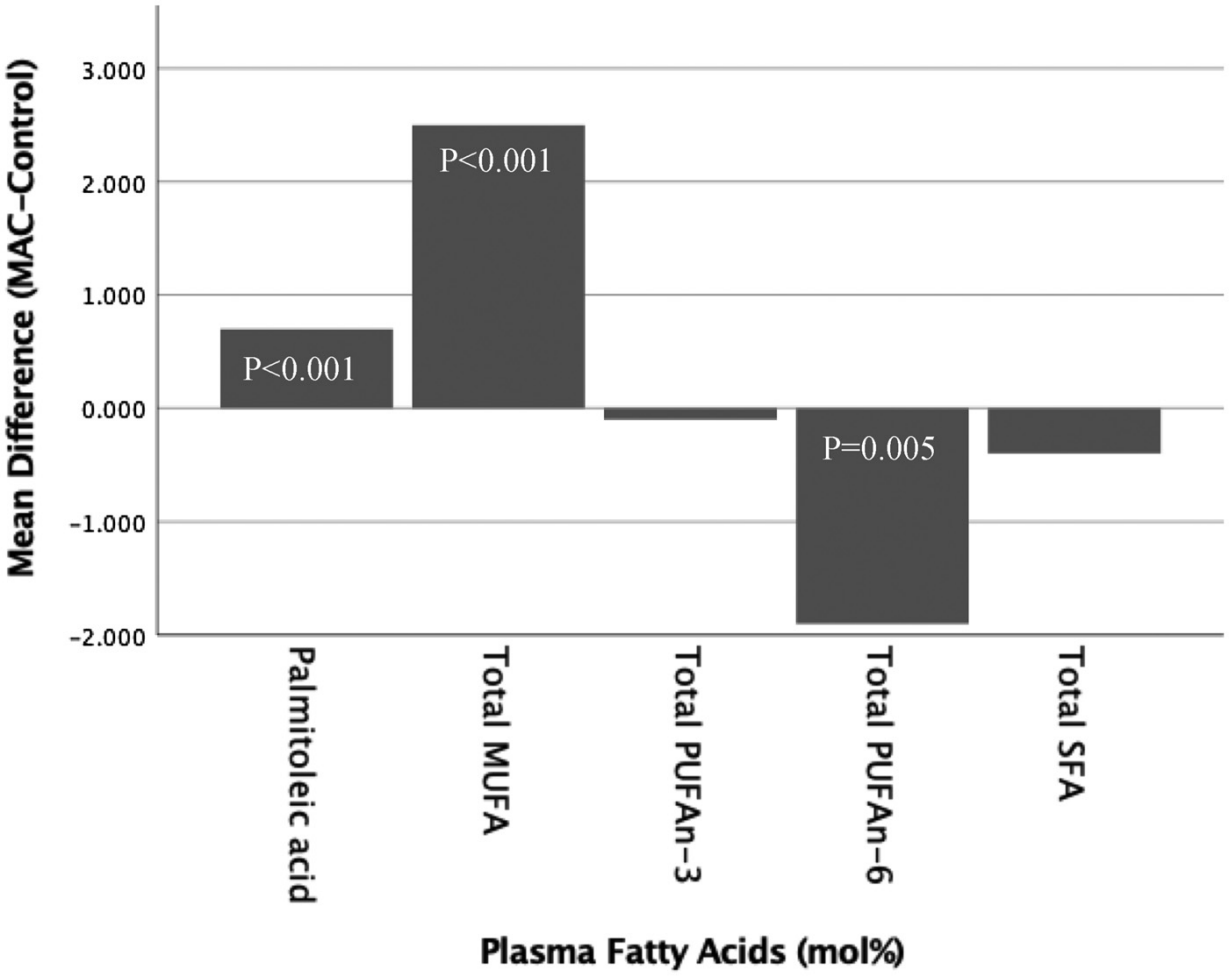
Plasma fatty acids presented as the mean difference between macadamia and control diets in mixed model analysis.

Plasma fatty acid levels confirmed the higher MUFA intake during the macadamia nut phase, with a mean increase in MUFA of 2⋅5 % (*P* < 0⋅001; 95 % CI 1⋅7 %, 3⋅4 %) (Fig. 1). This was partially accounted for by a higher plasma 16:1n-7 palmitoleic acid with macadamia nuts, which increased 26 % compared to control (absolute increase 0⋅7 %; *P* < 0⋅001; 95 % CI 0⋅5 %, 1⋅0 %). Both *n*-6 PUFA and *n*-3 PUFA made up a lower percentage of plasma fatty acids during the nut phase, with plasma *n*-6 PUFA significantly lower (mean difference −1⋅9 %; *P* = 0⋅005; 95 % CI −3⋅3, −0⋅6).

**Fig. 2.**
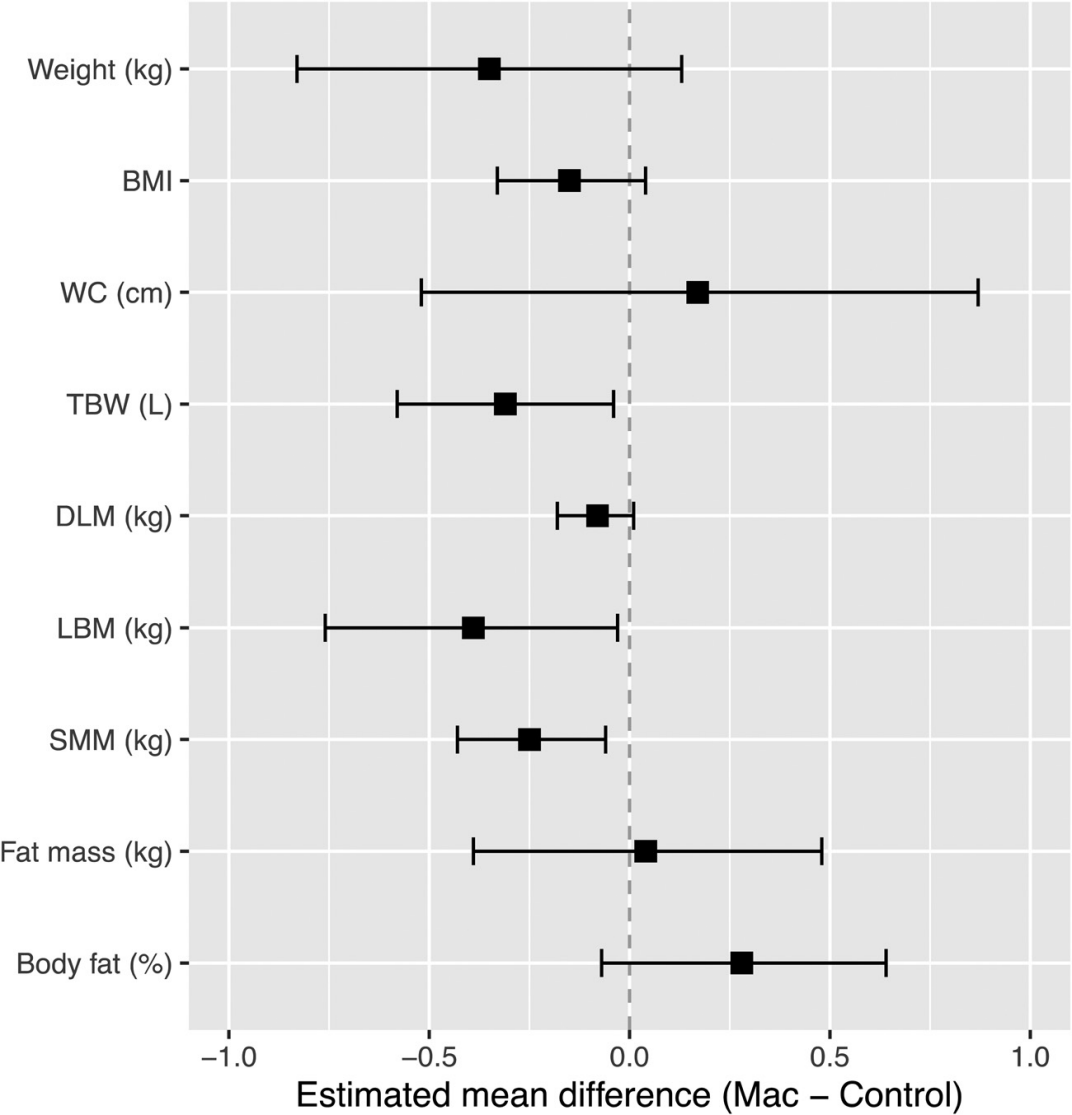
Anthropometric outcomes presented as the mean difference between macadamia and control diets in mixed model analysis.

Compared to the control, the consumption of macadamia nuts led to a small mean weight reduction of 350 g (NS; Fig. 2 and Supplementary Table S1). BMI, WC, BFM (kg) and % body fat demonstrated minimal differences with macadamia nuts, but significant small reductions were seen in SMM, LBM and TBW. SMM and TBW may be considered overlapping components of LBM.

**Fig. 3.**
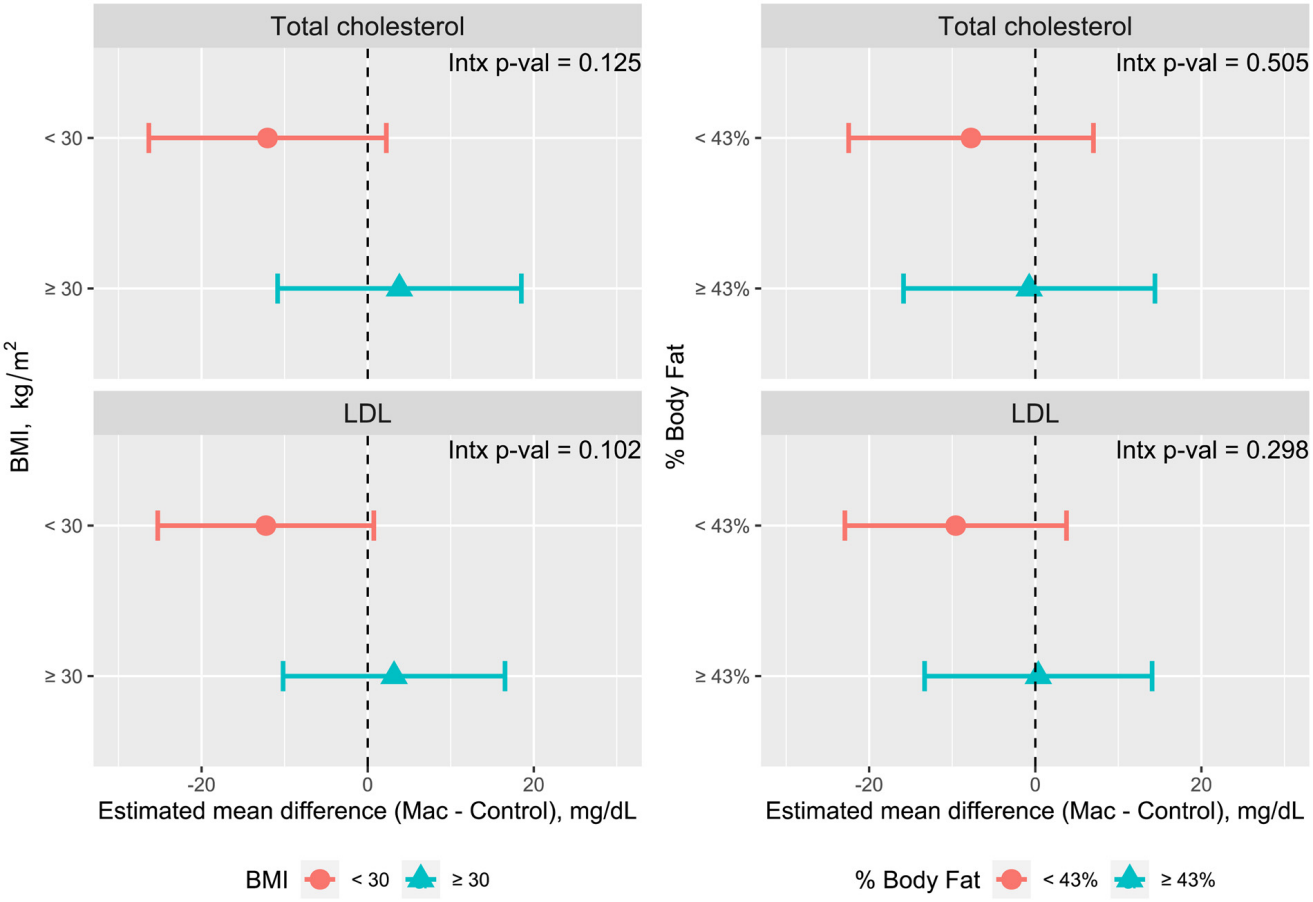
Total cholesterol and LDL-C by adiposity subgroups, presented as the mean difference between macadamia and control diets in mixed model analysis. Intx, interaction.

Consumption of macadamia nuts led to non-significant reductions of TC, LDL-C and Apo B compared to the control (Table 3). TC was reduced by 2⋅1 % and LDL-C by 4 % *v*. control. The components of metabolic dyslipidemia (HDL-C, TAG, sdLDL) and oxLDL were not significantly changed with macadamia nut consumption, nor did we observe differences in the glycaemic parameters, plasma glucose, insulin and HOMA2-IR (Table 3). Subgroup analyses of total and LDL-C by adiposity measures, including BMI (25–29⋅99 *v*. ≥30 kg/m^2^), median WC (<108 cm, ≥108 cm) and median % body fat (<43 %, ≥43 %), showed non-significant trends towards greater lowering of TC and LDL-C among participants with lower adiposity measures (Fig. 3 and Supplementary Table S2). Glucose, insulin and HOMA2-IR did not vary with adiposity (data not shown).

**Table 3.** Comparison of plasma lipid and glycaemic parameters during macadamia nut and control diets^a^

	MAC		Control		MAC–Control			
	Mean	sem	Mean	sem	Mean difference	se	*P*-value	95 % CI of difference
TC, mg/dL	197⋅1	5⋅0	201⋅4	5⋅0	−4⋅3	5⋅1	0⋅406	−14⋅8, 6⋅1
LDL-C, mg/dL	114⋅2	4⋅9	119⋅0	4⋅9	−4⋅7	4⋅7	0⋅320	−14⋅3, 4⋅8
HDL-C, mg/dL	56⋅4	1⋅8	56⋅6	1⋅8	−0⋅3	0⋅9	0⋅780	−2⋅2, 1⋅6
TAG, mg/dL	132⋅7	9⋅3	129⋅3	9⋅3	3⋅4	7⋅8	0⋅663	−12⋅4, 19⋅2
sdLDL, mg/dL	42⋅5	2⋅3	41⋅1	2⋅3	1⋅4	2⋅1	0⋅529	−3⋅0, 5⋅7
oxLDL, ng/mL	59⋅7	4⋅4	57⋅5	4⋅4	2⋅2	2⋅3	0⋅343	−2⋅4, 6⋅8
Glucose, mg/dL^b^	102⋅7	8⋅8	102⋅4	7⋅7	0⋅34	0⋅7	0⋅64	−1⋅2, 1⋅9
Insulin, IU/mL^b^	13⋅6	6⋅3	12⋅7	6⋅8	1⋅1	0⋅1	0⋅10	1⋅0, 1⋅2
HOMA2_IR	1⋅6	0⋅7	1⋅5	0⋅8	1⋅1	0⋅1	0⋅10	1⋅0, 1⋅2

MAC, macadamia nut diet; sem, standard error of the mean; se, standard error; CI, confidence interval; TC, total cholesterol; sdLDL, small dense low-density lipoprotein; oxLDL, oxidised low-density lipoprotein.

a Mixed model regression controlled for sequence and period.

b Log-transformed with estimated marginal means back-transformed; Mac–Control refers to the ratio of treatment means after back transformation; se remains on a log scale.

## Discussion

In our study, daily consumption of macadamia nuts as 15 % of calories for 8 weeks by overweight and obese individuals in free-living conditions did not adversely affect weight or body composition indices. Stable body weight and adiposity occurred despite an average increased intake of 97 kcal/d. This is consistent with other nut studies, in which the addition of nuts leads to either no change or a reduction in body weight^(8–13)^. Possible mechanisms for the observed lack of weight gain with macadamia nuts include their high content of fibre and fat, both total and monounsaturated, a food matrix that reduces metabolisable energy, and effects on the microbiome^(47)^. However, the mean fibre intake was only minimally increased during the macadamia nut phase of our study and was likely not a major factor in weight maintenance.

MUFA and other unsaturated fats have a higher oxidation rate than saturated fats, with less propensity for storage in adipose tissue and associated weight gain. In a study comparing olive oil to cream, the rate of fat oxidation and the thermic effect of food were significantly higher at 5 h after consumption of the olive oil^(48)^. This is particularly relevant, as macadamia nuts contain approximately 47 % oleic acid^(18)^, the dominant fatty acid in olive oil. The food matrix of nuts also likely plays an important role by reducing metabolisable energy. Traditional Atwater calculations of the energy content of a food are obtained from the heat of combustion of the macronutrients present. However, this ignores the digestibility of the food. Recent studies of cashews, almonds, walnuts and pistachios have found the actual metabolisable energy of these nuts to be significantly less than predicted by macronutrient composition (16, 34, 21, and 5 % lower, respectively)^(47,49)^. Finally, the high fat content of macadamia nuts may increase satiety through effects on cholecystokinin, as seen with pine nut oil^(50)^.

Adding to the body of evidence that supports the inclusion of nuts for lowering cholesterol, we showed that adding macadamia nuts to the habitual diet in overweight/obese adults produced a small (although not significant) reduction in TC, LDL-C and Apo B that are of a magnitude similar to that seen with other nuts, especially for LDL-C. Recent meta-analyses of almond and walnut consumption found net LDL-C reductions of −5⋅83 mg/dl^(51)^ and −5⋅5 mg/dl^(52)^, just slightly greater than our −4⋅7 mg/dl reduction with macadamia nuts. A meta-analysis of sixty-one trials examining the effects of tree nuts on blood lipids found TC and LDL-C to be lowered by −4⋅7 and −4⋅8 mg/dl, nearly identical to our results^(7)^. The mean reduction in LDL-C of 4⋅7 mg/dl would be expected to lower CVD risk by approximately 2⋅4 % (~0⋅5 % per every 1 mg/dl decrease in LDL-C)^(53)^. This degree of lipid lowering is somewhat less than that in our pooled analysis of twenty-five trials of nut consumption (in which TC was reduced by 10⋅9 mg/dl and LDL-C by 10⋅2 mg/dl)^(14)^, and relatively modest compared to statin therapy, which may reduce LDL-C in the range of 1 mmol/l (38⋅7 mg/dl) or more^(53)^.

Importantly, we saw cholesterol lowering without a change in SFA intake. SFA intake was 1 % higher with macadamia nuts (NS). Instead, the macadamia diet increased MUFA intake 9⋅5 % from baseline and 11⋅5 % compared to the control diet. This is distinct from most prior trials of macadamia nuts: three of these were controlled feeding studies in which LDL-C cholesterol reductions of 4⋅5, 8⋅9 and 10⋅7 % were observed in association with 5–6 % reductions in SFA intake^(25,28,29)^. In the trial most like ours, macadamia nuts provided as 15 % of energy in a free-living environment for 4 weeks resulted in a 0⋅7 % increase in SFA intake and a 5⋅3 % reduction in LDL-C, findings nearly identical to ours^(27)^. In this study and ours, a mechanism for cholesterol lowering beyond SFA must be at work.

Possible mechanisms by which nuts, including macadamia nuts, lower cholesterol include associated weight loss, increased fibre intake, large amounts of unsaturated fat relative to SFA and the presence of phytosterols^(51,52)^. Macadamia nuts contain large amounts of β-sitosterol, a phytosterol that competes with cholesterol for absorption^(18,19)^. A meta-analysis of the LDL-C-lowering effects of plant sterols and stanols found that 0⋅6–3⋅3 g/d reduces LDL-C in a dose-dependent manner by 6–12 %^(54)^. Macadamia nuts contain 116 mg of phytosterols per 100 g (33 mg/oz)^(18)^, which resulted in an intake of 41–69 mg of phytosterols per day in our study. This is less than the gram amounts usually associated with large lipid reductions^(54,55)^, though it may have been a factor.

Another likely mechanism for the lipid-lowering effects of macadamia nuts may be attributed to direct effects of MUFA on cholesterol homeostasis through action on CD36 and CD36-mediated metabolism^(56)^. Possible effects of MUFA in this regard include a reduction in endogenous cholesterol synthesis by reducing expression of 3-hydroxy-3-methylglutaryl-CoA reductase and sterol regulatory element-binding protein 2, as well as stimulation of ACAT1 to increase LDLR expression^(56)^.

The palmitoleic acid in macadamia nuts does not appear to have had a discernable effect on blood lipids. The components of metabolic dyslipidemia were not significantly changed, as we would expect if palmitoleate were acting as a lipokine^(20–22)^. It may be that the lipokine effect of palmitoleate is disrupted in obese individuals, and this complexity should be explored in future studies.

The trend towards greater lipid-lowering (TC and LDL-C) in association with lower BMI and adiposity (as measured by % body fat and WC) is consistent with the findings of our pooled analysis of nut consumption of all types^(14)^. In our pooled analysis, the greatest reductions of TC and LDL-C were seen in those with normal weight (BMI < 25 kg/m^2^), followed by overweight participants, with the smallest but still significant reductions in LDL among those with obesity. Additional data supports this finding. A study comparing the traditional American diet with Step I and II diets (lower in total and saturated fat) in healthy adult men found significantly smaller reductions in TC and LDL-C with increasing body weight, WC and % body fat, as well as with fasting glucose, insulin and IR^(16)^. A similar phenomenon was seen in a study comparing the effects of a high-saturated fat diet (38 % fat, 20 % SAT) to a National Cholesterol Education Program (NCEP)-I diet (28 % fat, 10 % SAT) in normal and overweight men: the overweight men had significantly smaller reductions in total and LDL-C with the lower-fat diet^(17)^.

Greater lipid reductions in individuals with a lower adiposity have been most clearly related to IR that frequently occurs with visceral adiposity^(16,57)^. Our cohort did not have a high degree of IR (baseline mean HOMA2-IR 1.35)^(58)^, nor did we find that macadamia nuts induced a difference in IR or any interactions between IR and plasma cholesterol. Yet even early IR in the setting of abdominal obesity seems to increase production rates of VLDL, LDL and LDL-C in women^(59)^. Insulin is also important to the presence and function of hepatic LDL receptors (LDLR)^(60,61)^. In mice lacking hepatic insulin receptors, hepatic LDLRs are reduced by at least 90 %^(60)^, and in hepatic cell culture, the presence of insulin leads to increased LDL-C clearance^(61)^. It may be that even mild IR disrupts normal LDL-C production and clearance, reducing the responsiveness to lipid-lowering interventions.

Our study has several strengths, including the cross-over design and a high rate of compliance with the intervention confirmed with a serum biomarker. It is the longest study on macadamia nuts to date, and the free-living nature of the study provides external validity. The biggest limitation of the study was the small sample size. The study was powered to detect a difference in LDL-C of 10 mg/dl and may have been underpowered to detect significance at the level of changes observed. Additionally, while the plasma fatty acid results generally confirmed the results of the 24-h recalls of dietary intake, measurement error associated with the dietary intakes cannot be excluded.

In conclusion, macadamia nut intake in overweight and obese individuals modestly lowered TC and LDL-C in the context of no change in SFA intake and with no change in body weight, and the magnitude of lipid lowering was greater in participants with lower adiposity. These findings have several public health implications. First, they provide evidence that MUFA may directly benefit cholesterol metabolism, independent of SFA intake. Second, adiposity may be an effect modifier of the lipid-lowering effects of nuts. Nuts and other lipid-lowering dietary interventions appear to be less helpful in the setting of abdominal obesity, and weight loss should remain an important component of cholesterol-lowering therapy. Finally, macadamia nuts may be suggested as a nutrient-dense food even among the overweight/obese population without fear of weight gain.

## Supplementary material

For supplementary material accompanying this paper visit https://doi.org/10.1017/jns.2023.39. click here to view supplementary material

## References

[1] Guasch-Ferré M, Liu X, Malik VS, (2017) Nut consumption and risk of cardiovascular disease. J Am Coll Card 70, 2519–2532. 10.1016/j.jacc.2017.09.035 PMC5762129 29145952

[2] Liu G, Guasch-Ferré M, Hu LY, (2019) Nut consumption in relation to cardiovascular disease incidence and mortality among patients with diabetes mellitus. Circ Res 124, 920–929. 30776978 10.1161/CIRCRESAHA.118.314316 PMC6417933

[3] Fraser GE, Sabaté J, Beeson WL, (1992) A possible protective effect of nut consumption on risk of coronary heart disease. The Adventist Health Study. Arch Intern Med 152, 1416–1424. 1627021

[4] Ros E (2015) Nuts and CVD. Br J Nutr 113, S111–S120. 26148914 10.1017/S0007114514003924

[5] Estruch R, Ros E, Salas-Salvadó J, (2018) Primary prevention of cardiovascular disease with a Mediterranean diet supplemented with extra-virgin olive oil or nuts. N Engl J Med 378, e34. doi:10.1056/NEJMoa1800389. Epub 2018 Jun 13. PMID: 29897866. 29897866

[6] Aune D, Keum N, Giovannucci E, (2016) Nut consumption and risk of cardiovascular disease, total cancer, all-cause and cause-specific mortality: a systematic review and dose-response meta-analysis of prospective studies. BMC Med 14, 207. 27916000 10.1186/s12916-016-0730-3 PMC5137221

[7] Del Gobbo LC, Falk MC, Feldman R, (2015) Effects of tree nuts on blood lipids, apolipoproteins, and blood pressure: systematic review, meta-analysis, and dose-response of 61 controlled intervention trials. Am J Clin Nutr 102, 1347–1356. doi:10.3945/ajcn.115.110965. 26561616 PMC4658458

[8] Bes-Rastrollo M, Sabaté J, Gomez-Gracia E, (2007) Nut consumption and weight gain in a Mediterranean cohort: the SUN study. Obesity 15, 107–116. 17228038 10.1038/oby.2007.507

[9] Bes-Rastrollo M, Wedick NM, Martınez-Gonzalez MA, (2009) Prospective study of nut consumption, long-term weight change, and obesity risk in women. Am J Clin Nutr 89, 1913–1919. 19403639 10.3945/ajcn.2008.27276 PMC2683001

[10] Mozaffarian D, Hao T, Rimm EB, (2001) Changes in diet and lifestyle and long-term weight gain in women and men. N Engl J Med 364, 2392–2404. 10.1056/NEJMoa1014296 PMC3151731 21696306

[11] Flores-Mateo G, Rojas-Rueda D, Basora J, (2013) Nut intake and adiposity: meta-analysis of clinical trials. Am J Clin Nutr 97, 1346–1355. doi:10.3945/ajcn.111.031484. 23595878

[12] Babio N, Toledo E, Estruch R, (2014) Mediterranean diets, and metabolic syndrome status in the PREDIMED randomized trial. CMAJ 186, E649–E657. doi:10.1503/cmaj.140764. 25316904 PMC4234734

[13] Nishi SK, Viguiliouk E, Blanco Mejia S, (2021) Are fatty nuts a weighty concern? A systematic review and meta-analysis and dose-response meta-regression of prospective cohorts and randomized controlled trials. Obes Rev 22, e13330. doi:10.1111/obr.13330. Epub 2021 Sep 8. PMID: 34494363; PMCID: PMC9285885. 34494363 PMC9285885

[14] Sabaté J, Oda K & Ros E (2010) Nut consumption and blood lipid levels: a pooled analysis of 25 intervention trials. Arch Intern Med 170, 821–827. 20458092 10.1001/archinternmed.2010.79

[15] Godhia M & Naik N (2015) Altered lipid responses to dietary interventions in obesity. Curr Res Nutr Food Sci 3, 1–11.

[16] Lefevre M, Champagne CM, Tulley RT, (2005) Individual variability in cardiovascular disease risk factor responses to low-fat and low-saturated-fat diets in men: body mass index, adiposity, and insulin resistance predict changes in LDL cholesterol. Am J Clin Nutr 82, 957–963. doi:10.1093/ajcn/82.5.957. PMID: 16280425. quiz 1145-6. 16280425

[17] Jansen S, Lopez-Miranda J, Salas J, (1998) Plasma lipid response to hypolipidemic diets in young healthy non-obese men varies with body mass index. J Nutr 128, 1144–1149. https://0-doi-org.catalog.llu.edu/10.1093/jn/128.7.1144 9649598 10.1093/jn/128.7.1144

[18] USDA National Nutrient Database for Standard Reference Legacy Release, April 2018. https://ndb.nal.usda.gov/ndb/search/list?home=true

[19] Maguire LS, O'Sullivan SM, Galvin K, (2004) Fatty acid profile, tocopherol, squalene and phytosterol content of walnuts, almonds, peanuts, hazelnuts and the macadamia nut. Int J Food Sci Nutr 55, 171–178. 15223592 10.1080/09637480410001725175

[20] Frigolet ME & Gutiérrez-Aguilar R (2017) The role of the novel lipokine palmitoleic acid in health and disease. Adv Nutr 8, 173S–181S. 28096141 10.3945/an.115.011130 PMC5227969

[21] Cao H, Gerhold K, Mayers JR, (2008) Identification of a lipokine, a lipid hormone linking adipose tissue to systemic metabolism. Cell 134, 933–944. doi:10.1016/j.cell.2008.07.048. 18805087 PMC2728618

[22] Mozaffarian D, Cao H, King IB, (2010) Circulating palmitoleic acid and risk of metabolic abnormalities and new-onset diabetes. Am J Clin Nutr 92, 1350–1358. doi:10.3945/ajcn.110.003970. 20943795 PMC2980960

[23] Klop B, Elte JW & Cabezas MC (2013) Dyslipidemia in obesity: mechanisms and potential targets. Nutrients 5, 1218–1240. doi:10.3390/nu5041218. Published 2013 Apr 12. 23584084 PMC3705344

[24] Björnson E, Adiels M, Taskinen MR, (2017) Kinetics of plasma triglycerides in abdominal obesity. Curr Opin Lipidol 28, 11–18. doi:10.1097/MOL.0000000000000375. PMID: 27898581. 27898581

[25] Griel AE, Cao Y, Bagshaw DD, (2008) A macadamia nut-rich diet reduces total and LDL-cholesterol in mildly hypercholesterolemic men and women. J Nutr 138, 761–767. 18356332 10.1093/jn/138.4.761

[26] Hiraoka-Yamamoto J, Ikeda K, Negishi H, (2004) Serum lipid effects of a monounsaturated (palmitoleic) fatty acid-rich diet based on macadamia nuts in healthy, young Japanese women. Clin Exp Pharmacol Physiol 31, S37–S38. 15649284 10.1111/j.1440-1681.2004.04121.x

[27] Garg ML, Blake RJ & Wills RBH (2003) Macadamia nut consumption lowers plasma total and LDL cholesterol levels in hypercholesterolemic men. J Nutr 133, 1060–1063. 12672919 10.1093/jn/133.4.1060

[28] Curb JD, Wergowske G, Dobbs JC, (2000) Serum lipid effects of a high-monounsaturated fat diet based on macadamia nuts. Arch Intern Med 160, 1154–1158. 10789609 10.1001/archinte.160.8.1154

[29] Colquhoun DM, Humphries JA, Moores D, (1996) Effects of a macadamia nut enriched diet on serum lipids and lipoproteins compared to a low-fat diet. Food Australia 48, 216–221. Only abstract available.

[30] Bastien M, Poirier P, Lemieux I, (2014) Overview of epidemiology and contribution of obesity to cardiovascular disease. Prog Cardiovasc Dis 56, 369–381. doi:10.1016/j.pcad.2013.10.016. Epub 2013 Oct 24. PMID: 24438728. 24438728

[31] Academy of Nutrition and Dietetics Evidence Analysis Library “In overweight or obese adults, which predictive equation for estimating resting metabolic rate should be used?” Published 2014. https://www.andeal.org/template.cfm?template=guide_summary&key=4341 (accessed December 9, 2019).

[32] Rapid Assessment of Physical Activity (RAPA). University of Washington Health Promotion Research Center. https://depts.washington.edu/hprc/resources/products-tools/rapa/

[33] Allain CC, Poon LS, Chan CSG, (1974) Enzymatic determination of total serum cholesterol. Clin Chem 20, 470–475. 4818200

[34] Rieschlau P, Bernt E & Gruber WZ (1974) Enzymatic determination of total cholesterol in serum. Klin Chem Klin Biochem 12, 403–407. 4428856

[35] Trinder P (1969) Determination of glucose in blood using glucose oxidase with an alternative oxygen acceptor. Ann Clin Biochem 6, 24–25.

[36] Bucolo G & David H (1973) Quantitative determination of serum triglycerides by the use of enzymes. Clin Chem 19, 476–482. 4703655

[37] Stein MW (1965) Clinical Methods of Enzymatic Analysis, p. 117. Academic Press.

[38] Friedewald WT, Levy RI & Fredrickson DS (1972) Estimation of the concentration of low-density lipoprotein cholesterol in plasma, without use of the preparative ultracentrifuge. Clin Chem 18, 499–502. PMID: 4337382. 4337382

[39] Pagana KA & Pagana T (2021) Mosby's Diagnostic and Laboratory Test Reference, 15th ed., p. 571. St. Louis: Elsevier (Mosby).

[40] Babson AL (1991) The DPC cirrus IMMULITE automated immunoassay system. J Clin Immunoassay 14, 83–88.

[41] Folch J, Lees M & Sloane-Stanley GH (1957) A simple method for the isolation and purification of total lipid from animal tissues. J Biol Chem 226, 497–509. 13428781

[42] Agren JJ, Julkunen A & Penttila I (1992) Rapid separation of serum lipids for fatty acid analysis by a single aminopropyl column. J Lipid Res 33, 1871–1876. 1479296

[43] Matthan NR, Ip B, Resteghini N, (2010) Long-term fatty acid stability in human serum cholesteryl ester, triglyceride, and phospholipid fractions. J Lipid Res 51, 2826–2832. 20448292 10.1194/jlr.D007534 PMC2918465

[44] Matthan NR, Ooi EM, Van Horn L, (2014) Plasma phospholipid fatty acid biomarkers of dietary fat quality and endogenous metabolism predict coronary heart disease risk: a nested case-control study within the Women's Health Initiative observational study. J Am Heart Assoc 3, e000764. doi:10.1161/JAHA.113.000764. 25122663 PMC4310362

[45] The Homeostasis Assessment 2 (HOMA2) Calculator. https://www.dtu.ox.ac.uk/homacalculator/ (accessed July 2021).

[46] Wallace TM, Levy JC & Matthews DR (2004) Use and abuse of HOMA modeling. Diabetes Care 27, 1487–1495. 15161807 10.2337/diacare.27.6.1487

[47] Tindall AM, Petersen KS, Lamendella R, (2018) Tree nut consumption and adipose tissue mass: mechanisms of action. Curr Dev Nutr 2, nzy069. doi:10.1093/cdn/nzy069. PMID: 30488045; PMCID: PMC6252345. 30488045 PMC6252345

[48] Piers LS, Walker KZ, Stoney RM, (2002) The influence of the type of dietary fat on postprandial fat oxidation rates: monounsaturated (olive oil) vs saturated fat (cream). Int J Obes Relat Metab Disord 26, 814–821. doi:10.1038/sj.ijo.0801993. PMID: 12037652. 12037652

[49] Baer DJ & Novotny JA (2018) Metabolizable energy from cashew nuts is less than that predicted by Atwater factors. Nutrients 11, 33. doi:10.3390/nu11010033. PMID: 30586843; PMCID: PMC6356908. 30586843 PMC6356908

[50] Pasman WJ, Heimerikx J, Rubingh CM, (2008) The effect of Korean pine nut oil on in vitro CCK release, on appetite sensations and on gut hormones in post-menopausal overweight women. Lipids Health Dis 7, 10. doi:10.1186/1476-511X-7-10. PMID: 18355411; PMCID: PMC2322999. 18355411 PMC2322999

[51] Lee-Bravatti MA, Wang J, Avendano EE, (2019) Almond consumption and risk factors for cardiovascular disease: a systematic review and meta-analysis of randomized controlled trials. Adv Nutr 10, 1076–1088. doi:10.1093/advances/nmz043. PMID: 31243439; PMCID: PMC6855931. 31243439 PMC6855931

[52] Guasch-Ferré M, Li J, Hu FB, (2018) Effects of walnut consumption on blood lipids and other cardiovascular risk factors: an updated meta-analysis and systematic review of controlled trials. Am J Clin Nutr 108, 174–187. doi:10.1093/ajcn/nqy091. PMID: 29931130; PMCID: PMC6862936. 29931130 PMC6862936

[53] Cholesterol Treatment Trialists’ (CTT) Collaboration, Baigent C, Blackwell L, Emberson J, (2010) Efficacy and safety of more intensive lowering of LDL cholesterol: a meta-analysis of data from 170,000 participants in 26 randomised trials. Lancet 376, 1670–1681. doi:10.1016/S0140-6736(10)61350-5. Epub 2010 Nov 8. PMID: 21067804; PMCID: PMC2988224. 21067804 PMC2988224

[54] Ras RT, Geleijnse JM & Trautwein EA (2014) LDL-cholesterol-lowering effect of plant sterols and stanols across different dose ranges: a meta-analysis of randomised controlled studies. Br J Nutr 112, 214–219. doi:10.1017/S0007114514000750. Epub 2014 Apr 29. PMID: 24780090; PMCID: PMC4071994. 24780090 PMC4071994

[55] Abumweis SS, Barake R & Jones PJ (2008) Plant sterols/stanols as cholesterol lowering agents: a meta-analysis of randomized controlled trials. Food Nutr Res 52. doi:10.3402/fnr.v52i0.1811. Epub 2008 Aug 18. PMID: 19109655; PMCID: PMC2596710. PMC2596710 19109655

[56] Ulug E & Nergiz-Unal R (2020) Dietary fatty acids and CD36-mediated cholesterol homeostasis: potential mechanisms. Nutr Res Rev, 1–14. doi:10.1017/S0954422420000128. Epub ahead of print. PMID: 32308181. 32308181

[57] Capurso C & Capurso A (2012) From excess adiposity to insulin resistance: the role of free fatty acids. Vascul Pharmacol 57, 91–97. doi:10.1016/j.vph.2012.05.003. 22609131

[58] Gayoso-Diz P, Otero-González A, Rodriguez-Alvarez MX, (2013) Insulin resistance (HOMA-IR) cut-off values and the metabolic syndrome in a general adult population: effect of gender and age: EPIRCE cross-sectional study. BMC Endocr Disord 13. doi:10.1186/1472-6823-13-47. PMID: 24131857; PMCID: PMC4016563. PMC4016563 24131857

[59] Pont F, Duvillard L, Florentin E, (2002) Early kinetic abnormalities of apoB-containing lipoproteins in insulin-resistant women with abdominal obesity. Arterioscler Thromb Vasc Biol 22, 1726–1732. doi:10.1161/01.atv.0000032134.92180.41. PMID: 12377756. 12377756

[60] Biddinger SB, Hernandez-Ono A, Rask-Madsen C, (2008) Hepatic insulin resistance is sufficient to produce dyslipidemia and susceptibility to atherosclerosis. Cell Metab 7, 125–134. doi:10.1016/j.cmet.2007.11.013. PMID: 18249172; PMCID: PMC4251554. 18249172 PMC4251554

[61] Ramakrishnan G, Arjuman A, Suneja S, (2012) The association between insulin and low-density lipoprotein receptors. Diabetes Vascul Dis Res, 196–204. doi:10.1177/1479164111430243. 22278734

