# Supplementary material for: Macadamia nut effects on cardiometabolic risk factors: a randomised trial
Source: J Nutr Sci. 2023 May 8;12:e55. doi: 10.1017/jns.2023.39 (PMC10173088; doi:10.1017/jns.2023.39)
Supplement: Supplementary file 1 [file S2048679023000393sup001.docx]

| Supplementary Table 1. Adiposity outcomes for macadamia and control diets* | | | | | | | | | | |
| --- | --- | --- | --- | --- | --- | --- | --- | --- | --- | --- |
|  | Baseline | | Mac | | Control | | Mean Difference | | | |
|  | Mean | SD | Mean | SE | Mean | SE | Mac-Control | SED | 95% CI | P-value |
| Weight (Kg) | 83.1 | 14.1 | 83.7 | 2.5 | 84.1 | 2.5 | -0.35 | 0.2 | -0.8, 0.1 | 0.15 |
| BMI | 30.3 | 3.3 | 30.5 | 0.6 | 30.6 | 0.6 | -0.14 | 0.1 | -0.3, 0.04 | 0.13 |
| WC (cm) | 107.0 | 9.6 | 107.6 | 1.6 | 107.4 | 1.6 | 0.17 | 0.3 | -0.5, 0.9 | 0.61 |
| TBW (L) | 35.5 | 7.1 | 35.1 | 1.1 | 35.4 | 1.1 | -0.31 | 0.1 | -0.6, -0.04 | 0.03 |
| DLM (Kg) | 12.8 | 2.6 | 12.6 | 0.4 | 12.7 | 0.4 | -0.08 | 0.05 | -0.2, 0.01 | 0.09 |
| LBM (Kg) | 48.3 | 9.7 | 47.7 | 1.6 | 48.1 | 1.6 | -0.39 | 0.2 | -0.8, -0.03 | 0.03 |
| SMM (Kg) | 26.5 | 5.7 | 26.1 | 0.9 | 26.3 | 0.9 | -0.25 | 0.1 | -0.4 -0.1 | 0.01 |
| Fat Mass (Kg) | 34.8 | 8.0 | 36.1 | 1.5 | 36.0 | 1.5 | 0.04 | 0.2 | -0.4, 0.5 | 0.84 |
| Body Fat (%) | 41.9 | 5.9 | 43.0 | 1.0 | 42.7 | 1.0 | 0.28 | 0.2 | -0.01, 0.6 | 0.11 |
| MAC, Macadamia nut diet; SD, standard deviation; SE, Standard error; SED, Standard error of the difference; BMI, Body Mass Index; WC, Waist circumference; TBW, Total Body Water; DLM, Dry Lean Mass; LBM, Lean Body Mass; SMM, Skeletal Muscle Mass.  * Mixed model regression controlled for sequence and period.  Note:  LBM = DLM + TBW; LBM + Body Fat Mass = Weight | | | | | | | | | | |
